# Unconventional pairings of spin-orbit coupled attractive degenerate Fermi gas in a one-dimensional optical lattice

**DOI:** 10.1038/srep14863

**Published:** 2015-10-07

**Authors:** Junjun Liang, Xiaofan Zhou, Pak Hong Chui, Kuang Zhang, Shi-jian Gu, Ming Gong, Gang Chen, Suotang Jia

**Affiliations:** 1 State Key Laboratory of Quantum Optics and Quantum Optics Devices, Institute of Laser spectroscopy, Shanxi University, Taiyuan 030006, P. R. China; 2 Department of Physics and Center for Quantum Coherence, The Chinese University of Hong Kong, Shatin, N.T., Hong Kong, China

## Abstract

Understanding novel pairings in attractive degenerate Fermi gases is crucial for exploring rich superfluid physics. In this report, we reveal unconventional pairings induced by spin-orbit coupling (SOC) in a one-dimensional optical lattice, using a state-of-the-art density-matrix renormalization group method. When both bands are partially occupied, we find a strong competition between the interband Fulde-Ferrell-Larkin-Ovchinnikov (FFLO) and intraband Bardeen-Cooper-Schrieffer (BCS) pairings. In particular, for the weak and moderate SOC strengths, these two pairings can coexist, giving rise to a new phase called the FFLO-BCS phase, which exhibits a unique three-peak structure in pairing momentum distribution. For the strong SOC strength, the intraband BCS pairing always dominates in the whole parameter regime, including the half filling. We figure out the whole phase diagrams as functions of filling factor, SOC strength, and Zeeman field. Our results are qualitatively different from recent mean-field predictions. Finally, we address that our predictions could be observed in a weaker trapped potential.

Ultracold atoms have become standard toolboxes for simulating fundamental physics with strong interactions^1^,^2^. Recently, these systems are used to mimic the spin-orbit coupling (SOC)^3^, which is one of the most intriguing interaction in nature. In particular, the one-dimensional (1D) SOC—the simplest non-Abelian gauge potential^4^—has been realized experimentally in fermionic ^40^K^5^,^6^,^7^ and ^6^Li^8^ atoms, by using a similar scheme achieved in bosonic ^87^Rb atom^9^. This remarkable progress opens an immediate possibility for exploring nontrivial quantum phases of degenerate Fermi gases^10^. Some intriguing phases, including the topological Bardeen-Cooper-Schrieffer (BCS)^11^,^12^,^13^,^14^,^15^,^16^,^17^,^18^ and topological Fulde-Ferrell-Larkin-Ovchinnikov (FFLO)^19^,^20^,^21^ phases, have been revealed. The defects in these topological phases are expected to host self-Hermitian Majorana fermions, which are the major building blocks for achieving topological quantum computation^22^. The basic picture for realizing these nontrivial topological superfluids is that SOC, Zeeman field, and *s*-wave interaction can induce triplet *p*-wave pairing, when the chemical potential just occupies one single band^23^,^24^,^25^. In this regard, understanding the true pairing(s) in the spin-orbit-coupled systems is essential for achieving these novel phases.

All the previous predictions, both in free space and optical lattice, are demonstrated in the framework of mean-field theory^11^,^12^,^13^,^14^,^15^,^16^,^17^,^18^,^19^,^20^,^21^. In the detailed calculations, the pairing is simply assumed to take place between two fermions with a total center-of-mass momentum *Q*, which serves as a parameter to minimize the total free energy. *Q* = 0 and *Q* ≠ 0 correspond to the BCS and FFLO pairings, respectively. This fundamental picture is also widely used even in 1D systems^21^,^26^,^27^. In fact, in 1D the effect of quantum fluctuation becomes significant and the mean-field results are, in principle, unreliable^28^. This means that the true pairings in this new platform need to be examined more seriously, which is, however, still lacking. This work is devoted to addressing this fundamental issue in a 1D spin-orbit coupled optical lattice, using a state-of-the-art density matrix renormalization group (DMRG) method^29^,^30^.

Our numerical results demonstrate that the relevant physics in this model is completely modified by the SOC-induced triplet pairing^23^,^24^,^25^. (I) When both bands are partially occupied, the SOC can lead to a strong competition between the interband FFLO and intraband BCS pairings, due to the induced momentum-dependent spin polarizations. (II) For the weak and moderate SOC strengths, these two pairings can coexist, leading to a new phase called the FFLO-BCS phase. This new phase is characterized by a unique three-peak structure in pairing momentum distribution. (III) For the strong SOC strength, the system is dominated by the intraband BCS pairing in the whole parameter regime, including the half filling. (IV) We figure out the whole phase diagrams as functions of filling factor, SOC strength, and Zeeman field, in terms of the properties of pairing correlations in both real and momentum spaces. All the results predicted are qualitatively different from the recent mean-field predictions^27^. (V) Finally, we address the effect of the trapped potential on pairing correlations and local density. We show that our predictions could be observed in a weaker trapped potential, which is easily prepared in experiments.

## Results

### Model and Hamiltonian

We consider the following 1D Fermi-Hubbard model with a synthetic SOC^26^,^27^:


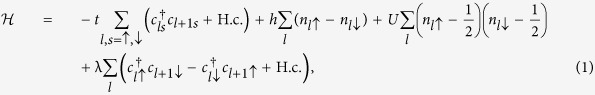


where 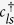 and 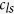 are the creation and annihilation operators, with spin 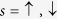 (encoded by the hyperfine states), at lattice site *l*, 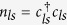 is the number operator, *t* is the spin-independent hopping, *h* is the Zeeman field along *z* direction, *U* is the on-site attractive interaction, λ is the SOC strength, and H.c. denotes the Hermitian conjugate.

Recently, the spin-orbit coupled Bose-Einstein condensate in a 1D optical lattice has been realized experimentally^31^. Using a similar technique, the Hamiltonian (1) could also be achieved in 1D degenerate Fermi gases^32^,^33^. Moreover, the corresponding parameters can be tuned widely. For example, the 3D optical lattice can be prepared by the interference of three pairs of counter-propagating laser beams^34^. The corresponding periodic potential is *V*_lattice_ = *V*_0_ cos^2^ (*k*_*w  *_*x*) + *V*_0_ cos^2^ (*k*_*w*_*y*) + *V*_0_ cos^2^ (*k*_*w*_*z*), where *V*_0_ is the lattice depth, *k*_*w*_ = λ_*w*_/2*π* is the wave vector, and λ_*w*_ is wavelength. By further using a large harmonic transverse confinement 
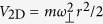
 in 3D optical lattice, i.e., the 2D harmonic potential frequency *ω*_^_ is far larger than the trapped frequency *ω*_*z*_ along the weakly-confining axis, the required 1D optical lattice can be generated^32^,^33^. In such case, the 1D effective interaction is described by^35^,^36^


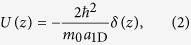


with the 1D *s*-wave scattering length


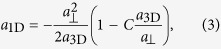


where 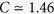, 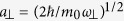, *a*_3D_ is the 3D *s*-wave scattering length, and *m*_0_ is the atomic mass. Equations (2) and (3) show that the 1D on-site attractive interaction can be tuned by Feshbach resonance^37^. In addition, for ^40^K^5^ or ^6^Li^8^ systems, two spin states are chosen respectively as 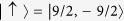 and 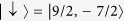, or 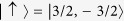 and 
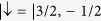
. By using a pair of counter-propagating Raman lasers, the 1D SOC in the Hamiltonian (1) can also be realized^5^,^6^,^7^,^8^,^9^. Moreover, the SOC strength can be tuned through a fast and coherent modulation of the Raman beams^38^; see also recent experiment^39^. For a typical optical lattice, the strong SOC strength, λ ∼  *t*, can be achievable^40^.

Since the effect of quantum fluctuation in 1D becomes significant, here we perform a state-of-the-art DMRG method to discuss the Hamiltonian (1). Notice that the similar Hamiltonian has been discussed by means of the same method^41^. In their work, they focused on the effect of the many-body interaction on topological phase and related Majorana fermions. In their calculation, the pairing term is preassigned to be the BCS pairing, i.e., no FFLO pairing can be driven from the many-body interaction. Here we mainly explore fundamental pairings induced by SOC, including the FFLO and BCS pairings.

**Figure 1:**
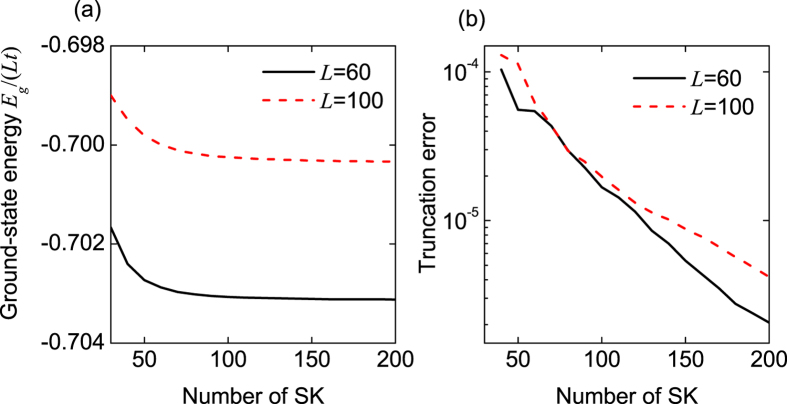
(a) The scaled ground-state energy *E*_*g*_/(*Lt*) and (b) the truncation error as functions of the number of states kept (SK). In all subfigures, *n* = 1, λ/*t* = 0.16, and *h*/*t* = 1.5.

In the following calculations, the basic energy scale is chosen as *t* = 1, the on-site interaction is set to *U*/*t* = −4, and the lattice lengths are chosen as *L* = 60 and 100. In addition, the open boundary condition is taken into account and 20 sweeps are always used. In Fig. 1(a), we plot the scaled ground-state energy *E*_*g*_/(*Lt*) as a function of the number of states kept. It can be seen that the scaled ground-state energy*E*_*g*_/(*Lt*) tends to a stable value, when increasing the number of states kept. This indicates that the number of states kept can be chosen as 150 per DMRG block. In Fig. 1(b), we show the truncation error as a function of the number of states kept. When the number of states kept is chosen as 150, the truncation error is smaller than 10^−5^, which is sufficient for the numerically-reliable results.

### Basic physical picture for unconventional pairings

**Figure 2:**
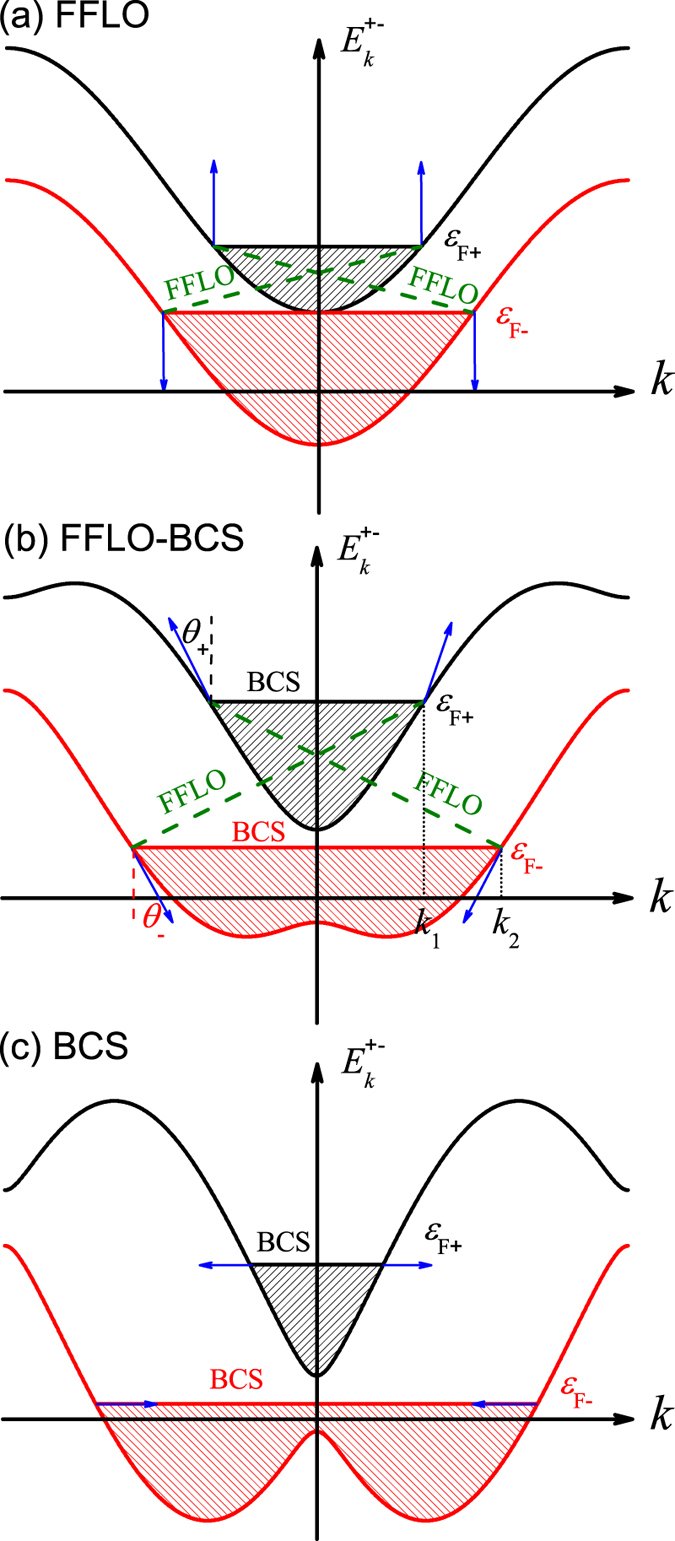
A schematic picture for illustrating unconventional pairings in a 1D optical lattice. (**a**) Without SOC, (**b**) the weak and moderate SOC strengths, and (**c**) the strong SOC strength. When both bands are partially occupied, there are two Fermi surfaces, denoted by *ε*_F±_, which give rise to four Fermi points ±*k*_1_ and ±*k*_2_ (see Supplementary Information). The corresponding spin-polarized angles at the two Fermi surfaces are defined as *θ*_±_, which are determined by the SOC strength and the Zeeman field; see Eq. (5). These polarizations are essential for describing the true pairings of the Hamiltonian (1). In (**a**), the spin is fully polarized along *z* direction (*θ*_±_ = 0), and only the interband FFLO pairing is thus formed. For the weak and moderate SOC strengths, *θ*_±_ are typically of the order of *π*/10 (see Supplementary Information). In such case, both the interband FFLO and intraband BCS pairings are allowed; see (**b**). More importantly, these two pairings can coexist, leading to a new phase called the FFLO-BCS phase. This new phase is characterized by a unique three-peak structure in pairing momentum distribution. For the strong SOC strength, the spin is almost polarized along *x* direction (*θ*_±_ ∼ *π*/2), and the intraband BCS pairing thus dominates; see (**c**).

Before proceeding, we first illustrate the basic physical picture of the Hamiltonian (1) with or without SOC. In the absence of SOC (λ/*t* = 0), the Hamiltonian 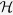 reduces to the well-studied Fermi-Hubbard model^42^,^43^, in which the Zeeman field breaks the degeneracy of each band [the breaking of symmetry from *SO*(4)^44^ at the half filling and otherwise *SU*(2) to 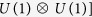; however, in both bands spin are still fully polarized along *z* direction. Consequently, the pairing can only be formed between two fermions at different bands, due to the interspecies interaction. This pairing gives rise to the well-known FFLO phase^45^,^46^, which can be observed at any nonzero population imbalance and has two nonzero center-of-mass momenta; see Fig. 2(a). However, this picture is completely modified by SOC, since it leads to momentum-dependent spin polarizations


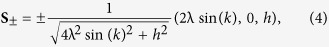


where ± denote the upper (+) and lower (−) bands. Since no spin polarizations can be found in the *y* component, the corresponding spin-polarized angles can be defined, using only one variable, as


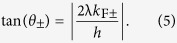


When both bands are partially occupied, the physics is extremely interesting. In this case, the SOC-induced triplet pairing can lead to the intraband BCS pairing, which can compete with the original interband FFLO pairing. This competition depends crucially on the spin-polarized angles *θ*_±_ at the Fermi points [in the mean-field level, the effective pairing is *p*-wave type with pairing strength 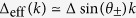
47], as shown below.

For a weaker SOC strength, i.e., 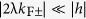, the spin is still fully polarized along *z* direction, and hence the spin-polarized angles *θ*_±_ ∼ 0. It indicates that the interband FFLO pairing dominates. When increasing the SOC strength, the spin will gradually polarize towards the *x* direction. For the weak and moderate SOC strengths, the spin-polarized angles *θ*_±_ are typically of the order of *π*/10 (see Supplementary Information). In such case, both the intraband BCS and interband FFLO pairings are allowed and govern simultaneously the true pairings of the Hamiltonian (1); see Fig. 2(b). For the strong SOC strength, the spin is almost polarized along *x* direction (*θ*_±_ ∼ *π*/2), and the intraband BCS pairing thus dominates; see Fig. 2(c). Therefore, we can expect a crossover from the interband FFLO pairing to the intraband BCS pairing, when the spin-polarized angles *θ*_±_ exceeds the critical values. We find that this transition is nontrivial because these two pairings can coexist in some parameter regimes; see below. Hereafter, all these pairings, which arise from strong competition between the two pairing channels induced by SOC and the Zeeman field, are called unconventional pairings. These unconventional pairings can lead to rich superfluid phases, which can be captured by considering pairing correlations in both real and momentum spaces.

### Pairing correlation in real space

The pairing correlation function in real space is defined as^48^,^49^,^50^,^51^


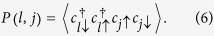


Without SOC, this pairing function can be used to identify the BCS and FFLO pairings. Physically, for the FFLO pairing, *P*(*l*, *j*) ∼ exp[*iQ*(*l* − *j*)], which oscillates in real space and exhibits two peaks at *k* = ±*Q* in momentum space^48^,^49^,^50^,^51^; see also discussions below. For the BCS pairing, *Q* = 0, and no oscillation can thus be found in real space. In the presence of SOC, we also use this function as an important tool (but not a unique tool) to identify the unconventional pairings of the Hamiltonian (1).

**Figure 3:**
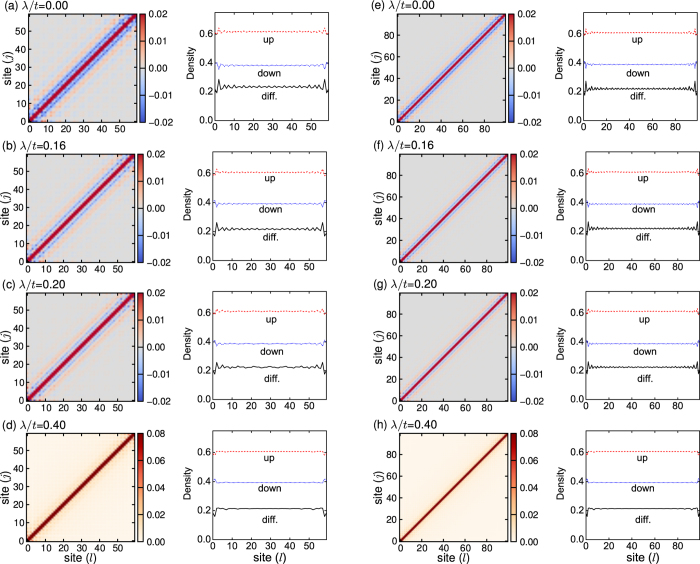
The pairing correlation functions *P*(*l*, *j*) and local densities *n*(*l*) for the different SOC strengths. Left two columns for *L* = 60 and right two columns for *L* = 100. In the local density, the solid line marks the local spin difference (diff.), which is defined as 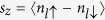. In all subfigures, *n* = 1 and *h*/*t* = 1.5.

**Figure 4:**
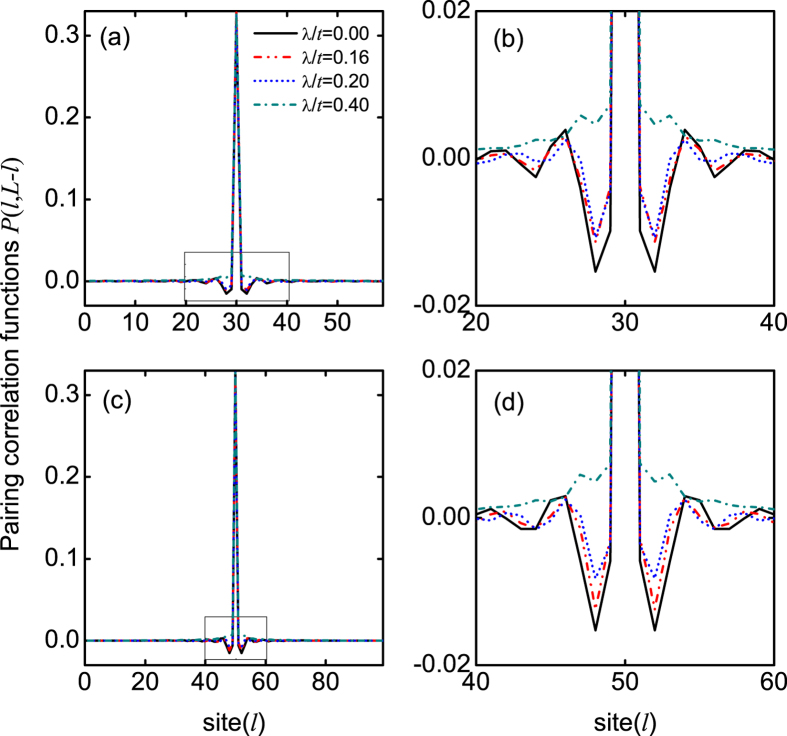
The off-diagonal pairing correlation functions *P*(*l*, *L* − *l*). (**a**) *P*(*l*, *L* − *l*) for the different SOC strengths, when *L* = 60, *n* = 1, and *h*/*t* = 1.5. (**b**) shows the zoomed images of the center 20 sites of (**a**). (**c**,**d**) are the same as those of (**a**,**b**), but with *L* = 100.

In Fig. 3, we plot the pairing correlation functions *P*(*l*, *j*) and the local densities *n*(*l*) for the different SOC strengths. Without SOC (λ/*t* = 0), the pairing correlation in real space exhibits strong oscillations in both magnitude and sign; see Fig. 3(a). Moreover, the local spin polarization also exhibits a similar oscillating behavior. This indicates the emergence of a FFLO phase^48^,^49^,^50^,^51^. For the moderate SOC strength [see, for example, λ/*t* = 0.16 and λ/*t* = 0.2 in Fig. 3(b,c)], the intraband BCS pairing increases, and has a strong competition with the interband FFLO pairing. In this case, the pairing correlations also have similar oscillating behaviors. For the strong SOC strength [see, for example, *λ*/*t* = 0.4 in Fig. 3(d)], the pairing correlation exhibits a power decay with respect to 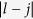 without node, and no obvious oscillation of spin polarization can be identified (the oscillation of spin polarization near the two ends is attributed to the finite-size effect). This means that a BCS phase emerges, as expected. In Fig. 4, we plot the off-diagonal pairing correlation functions *P*(*l*,  *L* − *l*) for the different SOC strengths. This figure also shows clearly the oscillations of the pairing correlation in real space, when the SOC strength is not very strong. This oscillation is gradually suppressed by increasing the SOC strength.

### Pairing momentum distribution

Although the pairing correlation functions at the weak and moderate SOC strengths exhibit the similar behaviors as those in the FFLO phase, their corresponding pairing momentum distributions *P*(*k*) have quite different behaviors. The pairing momentum distribution—the Fourier transformation of *P*(*l*, *j*)—is given by


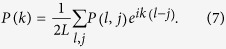


**Figure 5:**
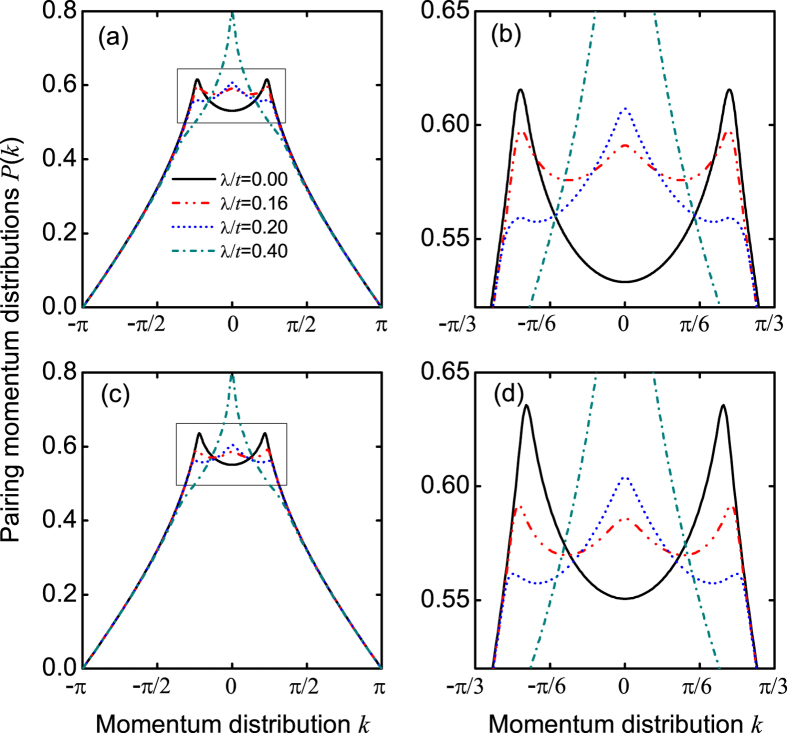
The pairing momentum distributions *P*(*k*). (**a**) *P*(*k*) for the different SOC strengths, when *L* = 60, *n* = 1, and *h*/*t* = 1.5. (**b**) shows the zoomed image of (**a**). (**c**,**d**) are the same as those of (**a**,**b**), but with *L* = 100.

Without SOC (λ/*t* = 0), the polarization angles *θ*_±_ = 0, and two nonzero center-of-mass momenta ±*Q*(≠0) can be found explicitly; see Fig. 5(a,b). This is a direct consequence of inversion symmetry in our model, *P*(*k*) = *P*(−*k*) is thus expected. The corresponding phase is referred as the FFLO phase^48^,^49^,^50^,^51^. When the SOC strength λ/*t* = 0.16, the polarization angles 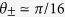 (see Supplementary Information). In such case, the dip at zero momentum of the pairing momentum distributions *P*(*k*) turns to a peak, while the other two peaks at ±*Q* change slightly (the detailed discussions are shown below). This indicates that the pairing momentum distribution *P*(*k*) has a unique three-peak structure, which demonstrates clearly that the intraband BCS and interband FFLO pairings can coexist. This result goes beyond the recent mean-field prediction^27^. We call the corresponding phase the *FFLO-BCS phase*. When the SOC strength λ/*t* = 0.20, the polarization angles 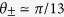, and the pairing mechanism is still similar to that of λ/*t* = 0.16. However, the peak of zero momentum is higher than that of nonzero momenta, which implies that the intraband BCS pairing is stronger than the interband FFLO pairing. For the strong SOC strength (see, for example, λ/*t* = 0.4), the polarization angles *θ*_±_ > *π*/10, and the intraband BCS pairing dominates. The corresponding phase is referred as the BCS phase, in which the pairing momentum distribution *P*(*k*) only has a peak at zero momentum. We need to emphasize that SOC affects significantly the pairing momentum distribution *P*(*k*) at the small momentum regime. For the large momentum regime, the system’s properties are determined mainly by the short-range interaction, and the pairing momentum distribution is thus unaffected by SOC; see also Fig. 5(a,c).

Since the pairing momentum distribution *P*(*k*) can be measured by the time-of-flight imaging^52^,^53^, the predicted three phases can be observed directly in experiments. The corresponding boundary between the FFLO and FFLO-BCS phases can be determined by


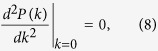


whereas the boundary between the FFLO-BCS and BCS phases can be determined by


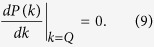


**Figure 6:**
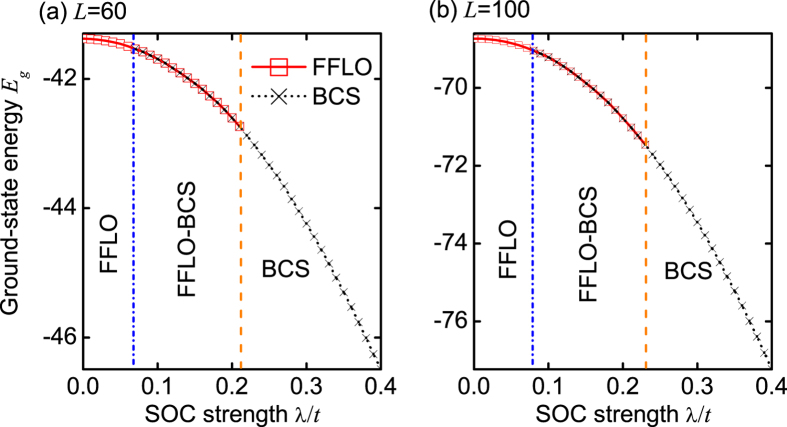
The ground-state energy *E*_*g*_/*t* as a function of the SOC strength. (**a**) *L* = 60 and (**b**) *L* = 100. In all subfigures, *n* = 1 and *h*/*t* = 1.5.

We now explain why the intraband BCS and interband FFLO pairings can coexist in the FFLO-BCS phase. In the presence of SOC, there are two bands (see Fig. 2), which contain spin-up and spin-down fermions. In the lower band, there are lots of fermions, which, however, become less in the upper band. Due to the existence of different spin-component fermions in the same band, the pairings can, in principle, be formed in the same or different bands, i.e., both the intraband BCS and interband FFLO pairings are allowed. For the small spin-polarized angles, the interband FFLO pairing is favored, while for the relative large spin-polarized angles, the intraband BCS pairing is favored. More importantly, the corresponding ground-state energies for both the intraband BCS and interband FFLO pairings are degenerate in the FFLO-BCS phase (see Fig. 6), which confirms the coexistence of these pairings.

**Figure 7:**
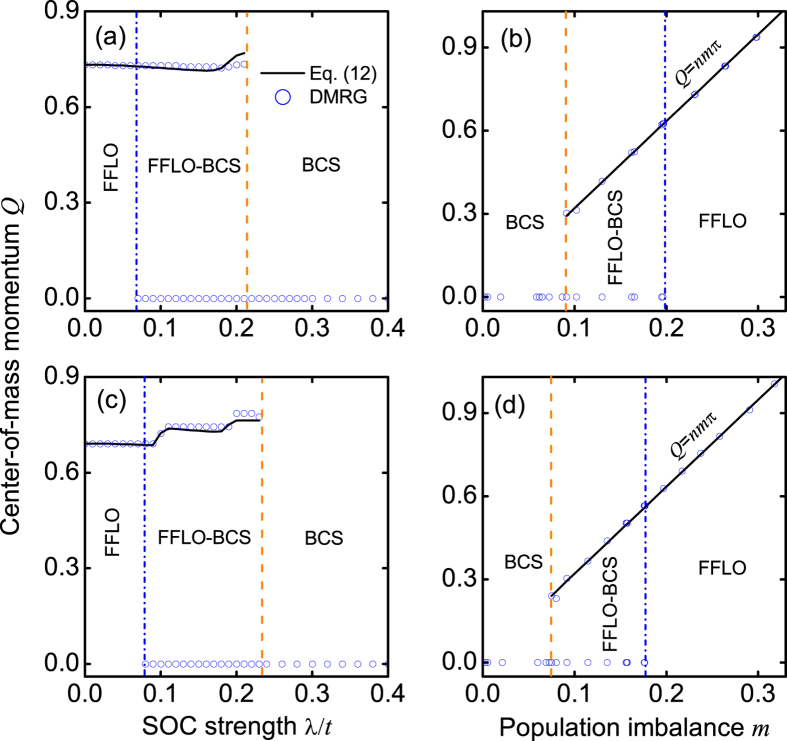
The center-of-mass momentum *Q*. (**a**) *Q*, which is derived respectively from the state-of-the-art DMRG calculations (Symbols) and analytical Eq. (12) (Solid line), as a function of the SOC strength, when *h*/*t* = 1.5, *n* = 1, and *L* = 60. In the analytical result, the population imbalance *m* is also obtained from the state-of-the-art DMRG calculations. (**b**) *Q* as a function of the population imbalance, when λ/*t* = 0.06, *n* = 1, and *L* = 60. (**c**,**d**) are the same as those of (**a**,**b**), but with *L* = 100.

In Fig. 7(a,c), we plot the center-of-mass momentum *Q* as a function of the SOC strength λ/*t*. Numerically, *Q* is determined by *dP*(*k*)/*dk* = 0 and *d*^2^*P*(*k*)/*dk*^2^ < 0. We find that *Q* is a non-monotonic function of the SOC strength λ/*t*. Here we develop a simple model to understand the relevant behavior. We assume that the Fermi points for two bands have momenta ±*k*_1_ and ±*k*_2_, respectively. These values are governed by the following equations:


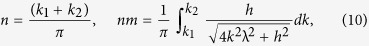


where *m* = (*N*_↑_ − *N*_↓_)/*N* is the experimentally-measurable population imbalance^54^,^55^. The center-of-mass momentum is determined by 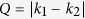.

For simplicity, we adopt the simplified model in free space, with which the analytical expression can be obtained perturbatively. We do not observe quantitatively modification of our conclusion by replacing *k* with sin(*k*) for a lattice model. For the weak SOC strength, we employ the Taylor expansion of Eq. (10) (up to the leading term) to obtain


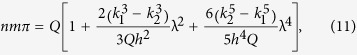


where *k*_1_ = (*nπ* − *Q*)/2 and *k*_2_ = (*nπ* + *Q*)/2. We assume the solution of *Q* has the following term


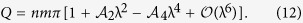


If letting the coefficient of λ^2^ and λ^4^ to be zero by the Taylor expansion of Eq. (11), we can immediately find


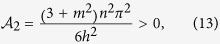



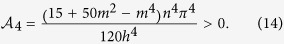


We find that Eqs. (12, 13, 14) can well describe the evolution of the center-of-mass momentum *Q* in the presence of a weak SOC; see Fig. 7(a,c). Moreover, without SOC (λ/*t* = 0), Eq. (12) reduces to the well-known result^48^,^49^,^50^,^51^: *Q* = *nmπ*.

**Figure 8:**
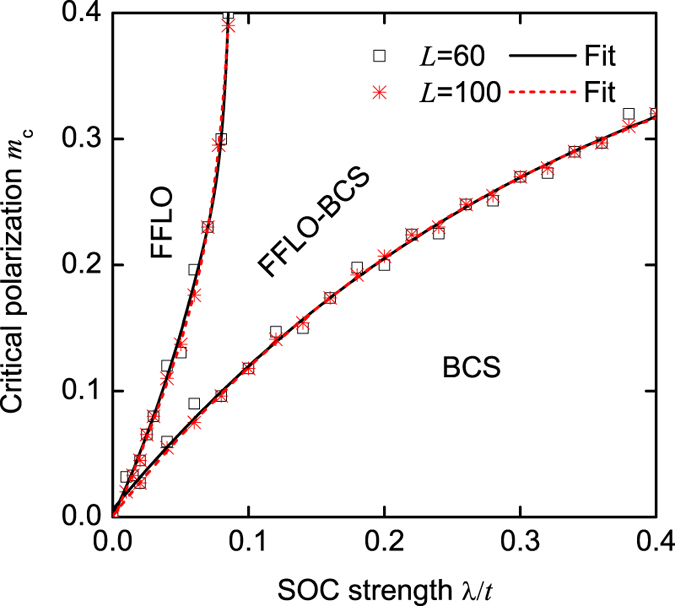
The critical population imbalance *m*_*c*_ as a function of the SOC strength. In this figure, *n* = 1.

From Eq. (12), we also see that without SOC, any nonzero population imbalance can give rise to the FFLO phase^48^,^49^,^50^,^51^. However, this basic conclusion is completely modified by SOC. In Fig. 7(b,d), we plot the center-of-mass momentum *Q* as a function of the population imbalance *m*, when the SOC strength λ/*t* = 0.06. We find that a finite population imbalance is required to realize the interband FFLO pairing in our model. Moreover, in both the FFLO-BCS and FFLO phases, *Q* = *nm*(λ)*π*, where *m*(λ) is obtained from the state-of-the-art DMRG calculations. In Fig. 8, we plot the relationship between the critical population imbalance *m*_*c*_ for the different phases and the SOC strength. Obviously, *m*_*c*_(λ = 0) = 0, as expected.

**Figure 9:**
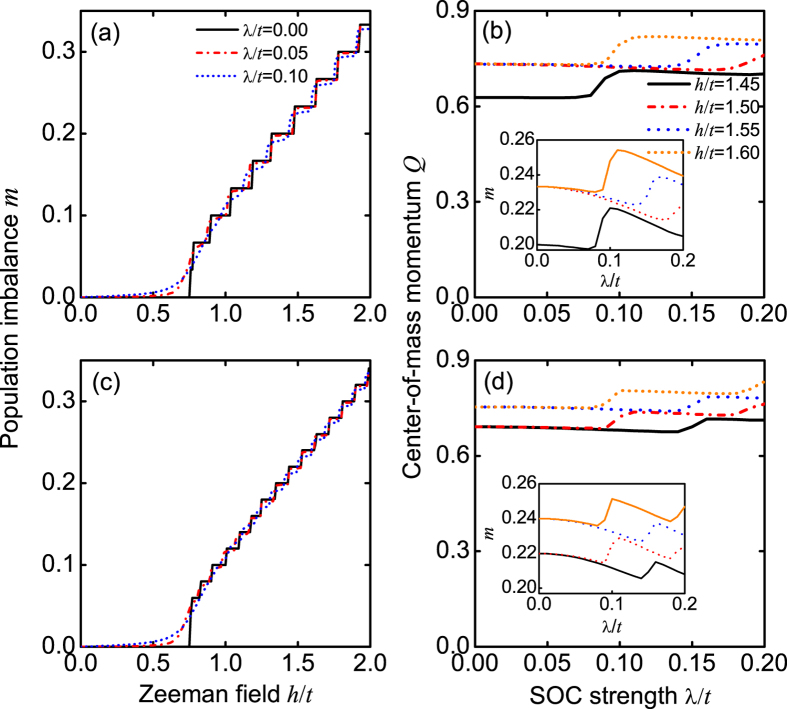
The population imbalance *m* and the center-of-mass momentums *Q* for the different Zeeman fields. (**a**) *m* as a function of the Zeeman field for the different SOC strengths, when *n* = 1 and *L* = 60.(**b**) *Q* as a function of the SOC strength for the different Zeeman fields, when *n* = 1 and *L* = 60. The inset of (**b**) shows *m* as a function of the SOC strength. (**c**,**d**) are the same as those of (**a**,**b**), but with *L* = 100.

In Fig. 9(a,c), we plot the population imbalances *m* as functions of the Zeeman field for the different SOC strengths, when *L* = 60 and *L* = 100. In the absence of SOC (λ/*t* = 0), the population imbalances *m* exhibit step behaviors for the finite-size lattice strengths. The corresponding step gap is given by Δ = 2/(*Ln*). When the lattice strength increases, this step gap becomes small and especially Δ → 0 for *L* → ∞. In the presence of SOC (λ/*t* ≠ 0), the finite-size step behaviors still exist but become smoother, since SOC can make fermions hop between the nearest-neighbor sites with spin flipping and thus has a strong effect on the population imbalances *m*. Similarly, the finite-size step behaviors also exist when the population imbalances *m* vary as the SOC strength; see the insets of Fig. 9(b,d). In terms of Eq. (12), we find straightforwardly that the finite-size step behaviors with respect to the SOC strength can lead to the similar behaviors of the center-of-mass momentum *Q*; see Fig. 9(b,d). Apart from the finite-size effect, the step behaviors of the center-of-mass momentum *Q* depend strongly on the Zeeman field and the SOC strength. For some parameter regimes, we find numerically that the corresponding steps become unobvious; see, for example, the red dash-dot line for *h*/*t* = 1.5 and *L* = 60 in Fig. 9(b).

**Figure 10:**
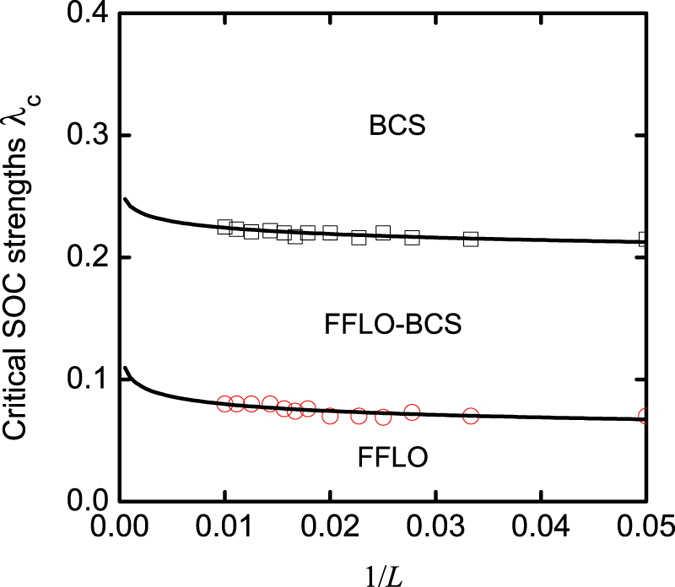
The critical SOC strengths λ_*c*_ as functions of the lattice length. In this figure, *n* = 1 and *h/t* = 1.5.

It should be pointed out that the boundary condition may influence the spin polarizations at the two ends; however, it does not affect our main predictions about spin polarizations in both real and momentum spaces, as demonstrated in Figs. 4 and 5 with *L* = 60 and *L* = 100. We also do not observe phase separation in the open boundary condition. So we can exclude the possibility of three peaks in the FFLO-BCS phase from the phase-separation effect. In Fig. 10, we plot the critical SOC strengths λ_*c*_, which govern the phase boundaries, as functions of the lattice length, when the Zeeman field *h*/*t* = 1.5 (the dash line of Fig. 12). In terms of this finite-size-scaling analysis, we find that when increasing the lattice length, our predicted FFLO-BCS phase, with a unique three-peak structure, still exists, although the center-of-mass momentum *Q* and the phase boundaries change slightly.

### Phase diagram

Having identified three superfluid phases, including the FFLO-BCS, FFLO, and BCS phases, we now figure out the corresponding phase diagram as a function of the filling factor, the Zeeman field, and the SOC strength. Numerically, the FFLO-BCS, FFLO, and BCS phases are characterized by three, two, and one peak(s) in the pairing momentum distribution *P*(*k*), respectively. In addition, when the fermions are fully polarized, i.e., *m* = 1, no pairing can occur. The corresponding phase is referred as the fully-polarized (FP) phase^32^. The boundary between the FFLO and FFLO-BCS phases can be determined by Eq. (8), whereas the boundary between the FFLO-BCS and BCS phases can be determined by Eq. (9).

**Figure 11:**
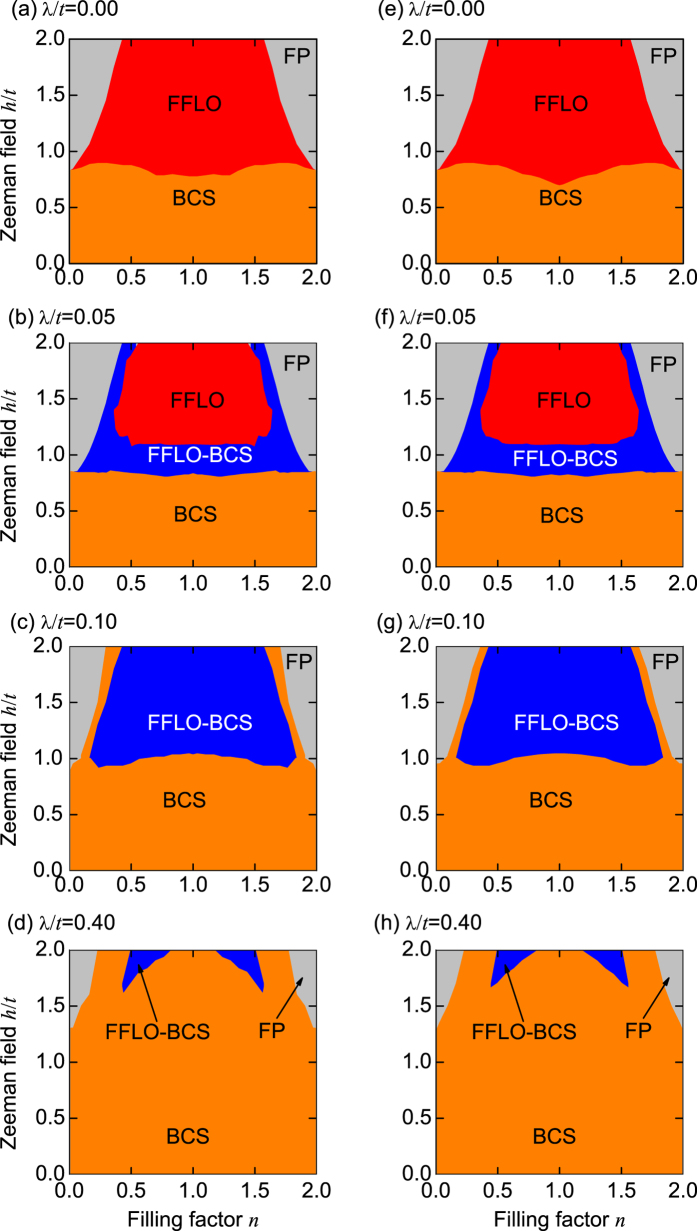
Phase diagrams in the *h* − *n* plane for the different SOC strengths. (**a**–**d**) *L* = 60 and (**e**–**h**) *L* = 100.

In Fig. 11, we plot the phase diagram in the *n* − *h* plane. In the absence of SOC (λ/*t* = 0), the FP, BCS, and FFLO phases can be found^42^; see Fig. 11(a). For the weak SOC strength (see, for example, λ/*t* = 0.05), the FFLO-BCS phase can be found, and the FFLO phase is suppressed; see Fig. 11(b). When the SOC strength λ/*t* = 0.1, the FFLO phase vanishes, and the FFLO-BCS phase is enhanced; see Fig. 11(c). For the strong SOC strength (see, for example, λ/*t* = 0.4), the FFLO-BCS phase almost disappears, and the BCS phase dominates in the whole parameter regime; see Fig. 11(d). These results demonstrate that for the weak SOC strength, a large regime for the FFLO phase can always be observed. However, for the strong SOC strength, the interband FFLO pairing are completely suppressed and the intraband BCS pairing always dominates in the whole parameter regime. This result is in contrast to that from mean-field prediction^27^, in which the FFLO phase always exists even for a stronger SOC strength (λ/*t* > 1).

In addition, all the phase diagrams in Fig. 11 are symmetric about the half filling (*n* = 1), which can be understood from the following particle-hole transformation:


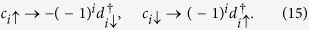


Under the transformation (15), we find (see Methods section)


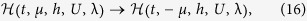


Here we have introduced a chemical potential *μ* to the original Hamiltonian (1), which equals exactly to zero at the half filling. Equation (16) demonstrates that the Hamiltonian (1) has the particle-hole symmetry. This symmetry ensures that the relevant physics in the low filling factor regime (*n* < 1) is identical to that in the high filling factor regime (*n* > 1), i.e., we have the observation in Fig. 11.

**Figure 12:**
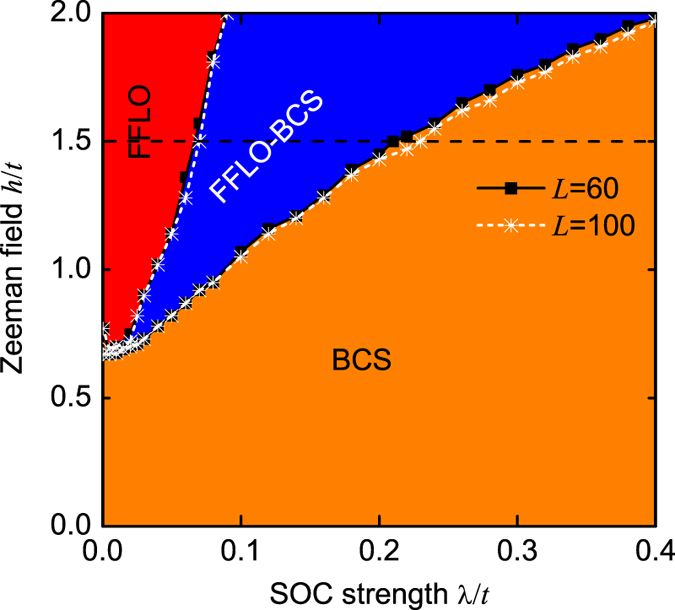
Phase diagram in the *h* − λ plane for the different lattice lengths *L* = 60 and *L* = 100. In this figure, *n* = 1.

Figure 12 shows the phase diagram in the *h* − λ plane at the half filling (*n* = 1), which further confirms that the interband FFLO pairing can be suppressed by the intraband BCS pairing. However, the situation for the FFLO-BCS phase is quite different. Since this phase requires not only an appropriate spin polarization but also a finite energy difference between *ε*_F±_ (see Supplementary Information), we see that it is more likely to be observed at a finite SOC strength and a stronger Zeeman field. Obviously, without the Zeeman field (*h*/*t* = 0), the spin is fully polarized along *x* direction (*θ*_±_ = *π*/2), and only BCS phase can be observed. In the presence of SOC, a stronger Zeeman field is thus required to bring the polarization along *z* direction (smaller than the critical polarization angle), so as to favor the interband FFLO pairing. We choose the results of *h*/*t* = 1.5 as an example to illustrate this point. In the absence of SOC (λ/*t* = 0), the polarization angles *θ*_±_ = 0, and we can only observe the FFLO phase. When λ/*t* < 0.07 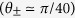, this interband FFLO pairing always dominates. However, when 0.07 < λ/*t* < 0.21 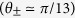, we find the FFLO-BCS phase. Finally, when λ/*t* > 0.21, the intraband BCS pairing dominates. Strikingly, we find that these critical angles are generally of the order of *π*/10, thus it is very easily to drive the FFLO phase to the BCS phase by a weak SOC.

## Discussion

**Figure 13:**
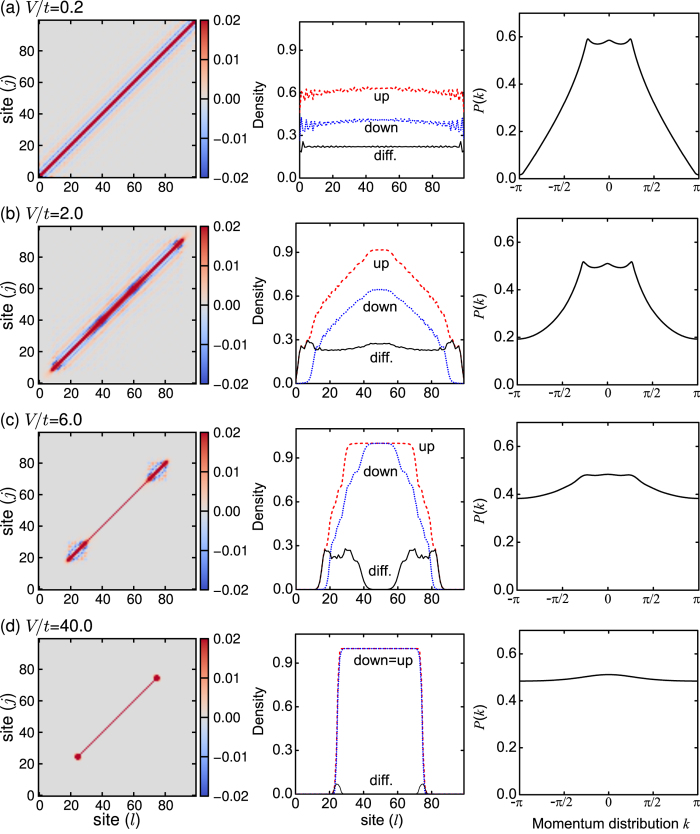
The pairing correlation functions *P*(*l*, *j*) (left column), the local densities *n*(*l*) (center column), and the pairing momentum distributions *P*(*k*) (right column) for the different trapped frequencies. In this figure, *h/t* = 1.5, *λ/t* = 0.16, and **L** = 100.

In real experiments, a harmonic trapped potential usually exists, and the Hamiltonian (1) should be added an extra term


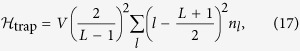


where *V* is the trapped frequency. In Fig. 13, we plot the pairing correlation functions *P*(*l*, *j*), the local densities *n*(*l*), and the pairing momentum distributions *P*(*k*) for the different trapped frequencies, when *h*/*t* = 1.5, λ/*t* = 0.16, and *L* = 100. The results for the other lattice length (such as *L* = 60) are similar and thus not plotted here.

Without the trapped potential, the system is located at the FFLO-BCS phase, in which the pairing correlation function *P*(*l*, *j*) has an oscillating behavior and the pairing momentum distribution *P*(*k*) has a unique three-peak structure; see Figs. 5 and 7. For a weaker trapped frequency (see, for example, *V*/*t* = 0.2), the oscillation of the pairing correlation function *P*(*l*, *j*) and especially three peaks of the pairing momentum distribution *P*(*k*) still exist; see Fig. 13(a). In addition, the corresponding density profile is almost the same as that without trapped potential; see Fig. 3(f). It means that no obvious phase separation in real space occurs. Thus, the predicted phase diagrams in Figs. 11 and 12, including the FFLO-BCS phase, also remain, although the corresponding phase boundaries change slightly. However, due to the existence of the trapped potential, the particle-hole symmetry of the inhomogeneous Hamiltonian 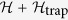 is broken, and the phase diagrams in Fig. 11 are not symmetric about the half filling (*n* = 1). When increasing the trapped frequency (see, for example, *V*/*t* = 2.0 and 6.0), the phase separation in real space occurs, since in this case the number of the fermions in the different sites is not same^48^,^50^,^51^,^54^,^55^,^56^,^57^. When the trapped frequency *V*/*t* = 2.0, the pairing correlation function *P*(*l*, *j*) exhibits an oscillation in 5 < *l* < 55, and the pairing momentum distribution *P*(*k*) has three peaks. However, the local density *n*(*l*) shows that the sites are fully polarized in two sides. It means that the FFLO-BCS and FP phases are mixed, and the system is thus located at the FFLO-BCS phase core with the FP phase wings; see Fig. 13(b). When the trapped frequency *V*/*t* = 6.0, the oscillation regime of the pairing correlation function *P*(*l*, *j*) turns into 10 < *l* < 20 and 40 < *l* < 50, and moreover, the pairing momentum distribution *P*(*k*) becomes smoother, i.e., no obvious peaks can be found. In addition, the local density *n*(*l*) shows the emergence of five phases, including the vacuum, FP, partly-polarized, metal, and band insulator phases (from left to center of the lattice); see Fig. 13(c). In the metal phase, all spin-down fermions can move freely in a uniform background of the spin-up fermions, and the band insulator is fully occupied by the spin-up and spin-down fermions^58^. Due to the phase separation in real space, the phase boundaries and the phase diagrams are hardly to be determined^48^. For a large trapped frequency (see, for example, *V*/*t* = 40.0), the physics is quite different, since in such case the term 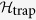 dominates in the inhomogeneous Hamiltonian. As a consequence, all fermions are forced to the center of the trap and there is only the band insulator without any moving fermions; see Fig. 13(d). From above discussions, it can be seen that our predictions could be observed for a weaker trapped potential, which is easily prepared in experiments.

In summary, we have shown, using the state-of-the-art DMRG calculations, that the true pairings in a 1D optical lattice can be completely modified by SOC, due to the induced triplet pairing. Especially, this system admits an exotic coexistence of the interband FFLO and intraband BCS pairings for the weak and moderate SOC strengths. However, for the strong SOC strength, the intraband BCS pairing always dominates, and the relevant physics is thus the BCS superfluid in the whole parameter regime. This yields a new picture to understand the true pairings in 1D spin-orbit coupled degenerate Fermi gases. The last conclusion (III) should be useful for searching the topological superfluids in this model. Finally, we have addressed the effect of the trapped potential on the pairing correlations and the local density. We have shown that our predictions could be observed in a weaker trapped potential, which is easily prepared in experiments.

## Methods

By means of the transformation (15), we find that the kinetic energy 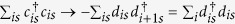, the chemical potential and Zeeman field 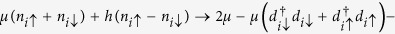

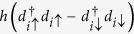
, the on-site attractive interaction 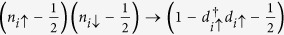

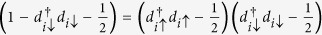
, and the SOC term 
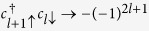

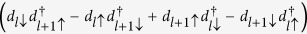

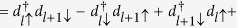

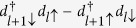
. As a result, we derive 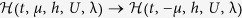, i.e., Eq. (16).

## Additional Information

**How to cite this article**: Liang, J. *et al.* Unconventional pairings of spin-orbit coupled attractive degenerate Fermi gas in a one-dimensional optical lattice. *Sci. Rep.* **5**, 14863; doi: 10.1038/srep14863 (2015).

## Supplementary Material

Supplementary Information
