# Supplementary material for: Unconventional pairings of spin-orbit coupled attractive degenerate Fermi gas in a one-dimensional optical lattice
Source: Sci Rep. 2015 Oct 7;5:14863. doi: 10.1038/srep14863 (PMC4595649; doi:10.1038/srep14863)
Supplement: Supplementary Information [file srep14863-s1.pdf]

Unconventional pairings of spin-orbit coupled attractive degenerate Fermi gas in a one-dimensional optical lattice

Junjun Liang,[1,*] Xiaofan Zhou,[1,*] Pak Hong Chui,[2] Kuang Zhang,[1]
Shi-jian Gu,[2] Ming Gong,[2,†] Gang Chen,[1,‡] and Suotang Jia[1]

[1]*State Key Laboratory of Quantum Optics and Quantum Optics Devices, Institute of Laser Spectroscopy, Shanxi University, Taiyuan 030006, China*

[2]*Department of Physics and Center for Quantum Coherence, The Chinese University of Hong Kong, Shatin, N.T., Hong Kong, China*

In Fig. 2 of main text, we have illustrated that when both bands are partially occupied, the true pairings depend strongly on the competition between the interband FFLO and intraband BCS pairings. Moreover, the spin-polarized angles $\theta_\pm$ are essential for describing the true pairings. In this supplementary material, we give the following supporting conclusions:

- Both bands are indeed partially occupied. This can be seen from the calculated k_1 and k_2, which are determined from Eq. (10) of main text. Generally, Q is determined by $|k_1 - k_2|$ in the FFLO phase. In the BCS phase, both k_1 and k_2 are nonzero. However, Q is zero because the interband pairing is not favorable in energy. The calculated k_1, k_2, and Q are shown in Table SI for $h/t = 0.5$, 1.0, and 1.5, respectively.

- The spin-polarized angles $\theta_\pm$ are essential for describing the interband FFLO and intraband BCS pairings. When $\theta_\pm$ are smaller (greater) then the critical values, the interband FFLO (intraband BCS) pairing dominates; however, in their intermediate regime, these two pairings can coexist. The calculated $\theta_\pm$ are presented in Table SI. At the half filling, we find $k_1 + k_2 = \pi$, and thus have

$$\mathbf{S}_+ \sim (2\lambda \sin(k_2), 0, h), \tag{S1}$$

$$\mathbf{S}_- \sim -(2\lambda \sin(k_1), 0, h). \tag{S2}$$

  We see immediately that $S_{1x} = S_{2x}$, but $S_{1z} = -S_{2z}$. It means that the spin-polarized angles satisfy the condition $\theta_+ + \theta_- = \pi$. For convenience, we redefine θ_- by $\pi - \theta_-$; see Fig. 2 of main text, and thus have $\theta_+ = \theta_-$. From the numerical results in Table SI, we see that the spin-polarized angles increase monotonically, when increasing the SOC strength. When $\theta_\pm$ exceed the critical values (typically the order of $\pi/10$), a discontinue change from the FFLO-BCS phase to the BCS phase occurs, and thus $Q = 0$, although $k_1 - k_2 \neq 0$.

---

* These authors contributed equally to this work
† skylark.gong@gmail.com
‡ chengang971@163.com

- The corresponding Fermi surface energies also provide an important evidence that both bands are partially occupied. The Fermi surface energies are calculated by

$$\varepsilon_{\mathrm{F}+} = -2t\cos(k_2) + \sqrt{4\lambda^2 \sin^2(k_2) + h^2}, \tag{S3}$$

$$\varepsilon_{\mathrm{F}-} = -2t\cos(k_1) - \sqrt{4\lambda^2 \sin^2(k_1) + h^2}, \tag{S4}$$

  where $\cos(k_1) + \cos(k_2) = 0$ and $\sin^2(k_1) = \sin^2(k_2)$. In this case, we immediately find that

$$\varepsilon_{\mathrm{F}+} = -\varepsilon_{\mathrm{F}-}. \tag{S5}$$

  The calculated Fermi surface energies are presented in Table SI and Fig. S1.

A possible question regarding these observations is that why we have four Fermi points ($\pm k_1$ and $\pm k_2$) or two Fermi surfaces ($\varepsilon_{\mathrm{F}+}$ and $\varepsilon_{\mathrm{F}-}$), although we just have one chemical potential. The reason is given as follows. Without the many-body interaction, all quantum states are occupied, when their energy is smaller than the chemical potential μ. That is, the four Fermi points *should* have the same energy. In this case, the spin imbalance may be very big for a large Zeeman splitting. This picture is totally modified for a strong attractive interaction U, which favors molecular bound state $|\uparrow\downarrow\rangle_i$, due to the on-site attractive interaction $Un_{i\uparrow}n_{i\downarrow}$. Imagining that $U \to -\infty$, then the ground state should be fully balanced, since all atoms with spin up form pairing with another atom with spin down, that is, $m = 0$. The price we have to pay is that we need four different Fermi points. This is exactly the basic idea in Fig. 2 of main text for a finite negative U. It is also important to see that even in the presence of SOC and Zeeman field, Q is still an implicit function of U. This is a basic conclusion obtained in previous literatures and it still holds here.

TABLE SI: Calculated data for Fig. 12 of main text, where λ is the SOC strength, h is the Zeeman field, m is the population imbalance from the state-of-the-art DMRG calculations, k_1 and k_2 are the momenta of the Fermi points predicted from Eq. (10) of main text, $Q = |k_1 - k_2|$, $\theta_\pm$ are the spin-polarized angles, and $\varepsilon_{F\pm}$ are the Fermi surface energies at the Fermi points. All the energies are in unit of t, while the momenta are in unit of $1/a$, where $a = 1$ is the lattice spacing. $U/t = -4$ and $L = 60$. $Q = 0.000$ in the sixth column means that the system is located at the BCS phase, although $k_1 - k_2 \neq 0$. In the last column, F, FB, and B correspond to the FFLO, FFLO-BCS, and BCS phases, respectively.

λ	h	m	k_1	k_2	Q	θ_+	θ_-	ε_{F+}	ε_{F-}	Phase
0.000	0.500	0.000	1.571	1.571	0.000	0.000	0.000	0.500	-0.500	B
0.020	0.500	0.000	1.571	1.570	0.000	0.027	0.027	0.501	-0.501	B
0.040	0.500	0.001	1.573	1.569	0.000	0.053	0.053	0.502	-0.502	B
0.060	0.500	0.002	1.575	1.567	0.000	0.080	0.080	0.506	-0.506	B
0.080	0.500	0.004	1.578	1.564	0.000	0.106	0.106	0.512	-0.512	B
0.100	0.500	0.006	1.581	1.561	0.000	0.133	0.133	0.518	-0.518	B
0.120	0.500	0.008	1.585	1.557	0.000	0.159	0.159	0.527	-0.527	B
0.140	0.500	0.010	1.588	1.553	0.000	0.185	0.185	0.538	-0.538	B
0.160	0.500	0.012	1.593	1.548	0.000	0.210	0.210	0.548	-0.548	B
0.180	0.500	0.014	1.598	1.544	0.000	0.235	0.235	0.562	-0.562	B
0.200	0.500	0.016	1.603	1.539	0.000	0.260	0.260	0.576	-0.576	B
0.220	0.500	0.018	1.609	1.533	0.000	0.285	0.285	0.590	-0.590	B
0.240	0.500	0.020	1.614	1.528	0.000	0.309	0.309	0.607	-0.607	B
0.260	0.500	0.022	1.621	1.521	0.000	0.333	0.333	0.622	-0.622	B
0.280	0.500	0.024	1.626	1.516	0.000	0.357	0.357	0.640	-0.640	B
0.300	0.500	0.025	1.633	1.509	0.000	0.380	0.380	0.656	-0.656	B
0.320	0.500	0.027	1.640	1.501	0.000	0.402	0.402	0.672	-0.672	B
0.340	0.500	0.029	1.648	1.494	0.000	0.425	0.425	0.689	-0.689	B
0.360	0.500	0.030	1.654	1.488	0.000	0.446	0.446	0.708	-0.708	B
0.380	0.500	0.032	1.662	1.479	0.000	0.467	0.467	0.725	-0.725	B
0.400	0.500	0.033	1.670	1.471	0.000	0.488	0.488	0.742	-0.742	B
0.000	1.000	0.100	1.728	1.414	0.314	0.000	0.000	0.687	-0.687	F
0.020	1.000	0.100	1.727	1.414	0.313	0.026	0.026	0.689	-0.689	F
0.040	1.000	0.099	1.728	1.414	0.314	0.053	0.053	0.690	-0.690	FB
0.060	1.000	0.102	1.732	1.409	0.323	0.079	0.079	0.686	-0.686	FB
0.080	1.000	0.115	1.756	1.386	0.370	0.104	0.104	0.645	-0.645	FB
0.100	1.000	0.113	1.749	1.392	0.000	0.130	0.130	0.664	-0.664	B
0.120	1.000	0.108	1.747	1.395	0.000	0.156	0.156	0.677	-0.677	B
0.140	1.000	0.104	1.739	1.403	0.000	0.182	0.182	0.702	-0.702	B
0.160	1.000	0.101	1.737	1.404	0.000	0.207	0.207	0.717	-0.717	B
0.180	1.000	0.099	1.737	1.405	0.000	0.232	0.232	0.731	-0.731	B
0.200	1.000	0.097	1.735	1.406	0.000	0.257	0.257	0.748	-0.748	B
0.220	1.000	0.096	1.737	1.405	0.000	0.282	0.282	0.760	-0.760	B
0.240	1.000	0.095	1.737	1.405	0.000	0.306	0.306	0.776	-0.776	B
0.260	1.000	0.095	1.737	1.405	0.000	0.329	0.329	0.794	-0.794	B
0.280	1.000	0.095	1.740	1.402	0.000	0.353	0.353	0.806	-0.806	B
0.300	1.000	0.095	1.745	1.396	0.000	0.375	0.375	0.814	-0.814	B
0.320	1.000	0.096	1.750	1.392	0.000	0.397	0.397	0.826	-0.826	B
0.340	1.000	0.096	1.755	1.386	0.000	0.419	0.419	0.836	-0.836	B
0.360	1.000	0.097	1.759	1.383	0.000	0.441	0.441	0.851	-0.851	B
0.380	1.000	0.098	1.765	1.377	0.000	0.461	0.461	0.862	-0.862	B
0.400	1.000	0.099	1.770	1.372	0.000	0.482	0.482	0.875	-0.875	B
0.000	1.500	0.233	1.937	1.204	0.733	0.000	0.000	0.783	-0.783	F
0.020	1.500	0.233	1.940	1.202	0.738	0.025	0.025	0.779	-0.779	F
0.040	1.500	0.232	1.936	1.205	0.731	0.050	0.050	0.787	-0.787	F
0.060	1.500	0.230	1.937	1.205	0.732	0.075	0.075	0.788	-0.788	F
0.080	1.500	0.228	1.932	1.209	0.723	0.099	0.099	0.800	-0.800	FB
0.100	1.500	0.225	1.924	1.218	0.707	0.124	0.124	0.820	-0.820	FB
0.120	1.500	0.222	1.922	1.220	0.702	0.149	0.149	0.830	-0.830	FB
0.140	1.500	0.219	1.919	1.222	0.697	0.174	0.174	0.840	-0.840	FB
0.160	1.500	0.216	1.919	1.223	0.696	0.198	0.198	0.848	-0.848	FB
0.180	1.500	0.215	1.919	1.222	0.697	0.222	0.222	0.855	-0.855	FB
0.200	1.500	0.223	1.933	1.209	0.725	0.244	0.244	0.837	-0.837	FB
0.220	1.500	0.223	1.935	1.206	0.000	0.267	0.267	0.842	-0.842	B
0.240	1.500	0.220	1.930	1.212	0.000	0.291	0.291	0.863	-0.863	B
0.260	1.500	0.217	1.932	1.210	0.000	0.314	0.314	0.871	-0.871	B
0.280	1.500	0.216	1.934	1.208	0.000	0.336	0.336	0.878	-0.878	B
0.300	1.500	0.217	1.937	1.204	0.000	0.357	0.357	0.885	-0.885	B
0.320	1.500	0.215	1.939	1.202	0.000	0.379	0.379	0.894	-0.894	B
0.340	1.500	0.214	1.939	1.203	0.000	0.400	0.400	0.909	-0.909	B
0.360	1.500	0.213	1.943	1.199	0.000	0.421	0.421	0.917	-0.917	B
0.380	1.500	0.212	1.946	1.195	0.000	0.440	0.440	0.925	-0.925	B
0.400	1.500	0.211	1.950	1.192	0.000	0.460	0.460	0.934	-0.934	B

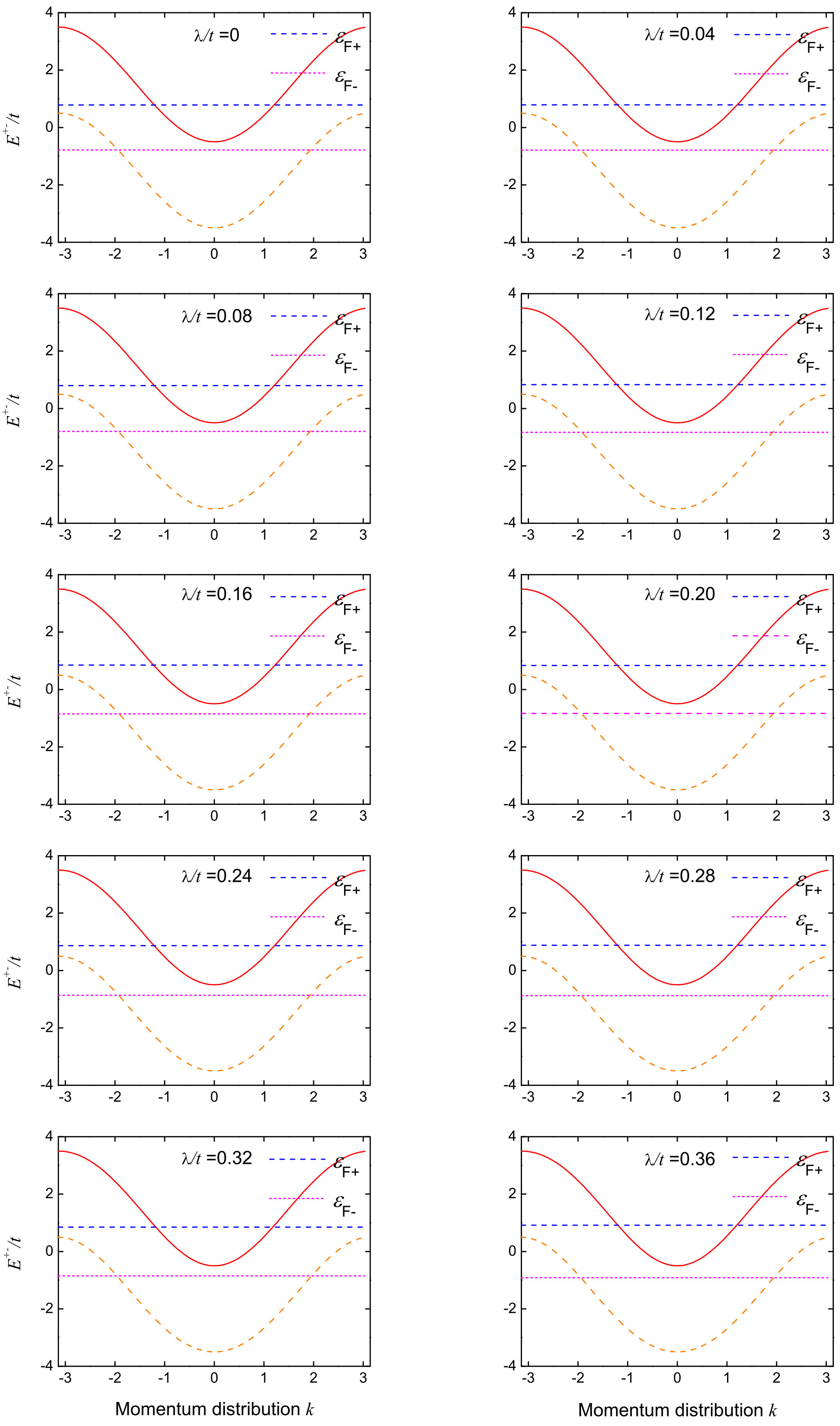

FIG. S1: (Color online). Band structure and filled regime for the different SOC strengths, when $h/t = 1.5$, $U/t = -4$, and $n = 1$. In this plot, $m(\lambda)$ is obtained directly from the state-of-the-art DMRG calculations. All the corresponding data are tabulated in Table SI for reference.
